# Transfer kinetics of perfluorooctane sulfonate from water and sediment to a marine benthic fish, the marbled flounder (*Pseudopleuronectes yokohamae*)

**DOI:** 10.1002/etc.2270

**Published:** 2013-07-11

**Authors:** Takeo Sakurai, Jun Kobayashi, Kyoko Kinoshita, Nozomi Ito, Shigeko Serizawa, Hiroaki Shiraishi, Jeong-Hoon Lee, Toshihiro Horiguchi, Hideaki Maki, Kaoruko Mizukawa, Yoshitaka Imaizumi, Toru Kawai, Noriyuki Suzuki

**Affiliations:** † National Institute for Environmental StudiesTsukuba, Japan; ‡ Faculty of Environmental & Symbiotic Sciences, Prefectural University of KumamotoKumamoto, Japan; § Southeast Sea Fisheries Research Institute, National Fisheries Research and Development InstituteTongyeong, Korea; ∥ Institute of Symbiotic Science and Technology, Tokyo University of Agriculture and TechnologyFuchu, Japan; # Research Fellow of the Japan Society for the Promotion of ScienceTokyo, Japan

**Keywords:** Bioconcentration, Persistent organic pollutants, Respiratory uptake, Aquatic organism, Particle

## Abstract

The authors investigated the kinetics of transfer of perfluorooctane sulfonate (PFOS) from water, suspended sediment, and bottom sediment to a marine benthic fish, the marbled flounder (*Pseudopleuronectes yokohamae*). Fish were exposed in 3 treatments to PFOS in combinations of these exposure media for 28 d and then depurated for 84 d. A major part (37–66%) of PFOS in the fish was in the carcass (i.e., whole body minus muscle and internal organs). Three first-order-kinetic models that differed in exposure media, that is, 1) sum of dissolved and particulate phases and sediment; 2) dissolved phase, particulate phase, and sediment; and 3) dissolved phase only, were fitted to the data assuming common rate constants among the treatments. The uptake efficiency of dissolved PFOS at the respiratory surfaces was estimated to be 3.2% that of oxygen, and the half-life of PFOS in the whole body to be 29 d to 31 d. The better fit of models 1 and 2 and the values of the estimated uptake rate constants suggested that the PFOS in suspended and bottom sediments, in addition to that dissolved in water, contributed to the observed body burden of the fish. Based on an evaluation of several possible contributing factors to the uptake of PFOS from suspended and bottom sediments, the authors propose that further investigation is necessary regarding the mechanisms responsible for the uptake. *Environ Toxicol Chem* 2013;32:2009–2017. © 2013 The Authors. *Environmental Toxicology and Chemistry* Published by Wiley Periodicals, Inc., on behalf of SETAC. This is an open access article under the terms of the Creative Commons Attribution Non-Commercial License, which permits use, distribution, and reproduction in any medium, provided the original work is properly cited and is not used for commercial purposes.

## INTRODUCTION

Bioaccumulation of perfluorooctane sulfonate (PFOS) in fish is of interest because it is expected to accumulate in the aquatic environment due to its negligible vapor pressure and relatively high water solubility 1. It has been a focus of concern 2 because its persistence 1 has led to its worldwide detection in humans, wildlife, ambient waters, and aquatic sediments 1,3. Consumption of fish can be a major source of human exposure to PFOS 5. Understanding the uptake and depuration kinetics of PFOS in fish is essential for predicting PFOS concentrations in fish in the environment. Because PFOS is a surfactant present as an anion in most ambient water 1, its kinetics may differ from those of neutral chemicals 6. There has been a limited number of kinetic studies of PFOS in fish at environmentally relevant levels of exposure 7 or dealing with respiratory uptake efficiency. Examination of the distribution of PFOS among fish tissues, including the parts consumed by humans, will enhance our understanding of its toxicokinetics and will be useful for assessing human exposure to PFOS.

The transfer of chemicals to aquatic organisms in the marine environment deserves further investigation 8. Marine fish account for more than half of global fishery production and of our dietary calorie intake from aquatic organisms. However, most chemical-transfer studies have used freshwater aquatic organisms 9.

In the aquatic environment, bottom and suspended sediments may play an important role as sources of persistent chemicals for the water column and aquatic organisms 10,11. In fish, only chemicals in the dissolved phase are considered to be taken up accompanying respiration 13. Potential uptake of these chemicals from bottom and suspended sediments has been investigated 8,11, but quantitative kinetic evaluation of such uptake is limited, particularly in the case of fish.

The objective of the present laboratory study was to determine the uptake and depuration kinetics of PFOS in a marine benthic fish, the marbled flounder. Marbled flounder is an appropriate experimental species because it lives on and in sediment and is therefore susceptible to potential impacts of sediment on the transfer kinetics of chemicals from the aquatic environment. The fish is commonly caught and eaten in Japan. Pleuronectiform fish, including flounders and soles, are distributed and eaten worldwide. We focused particularly on uptake efficiency via the respiratory surfaces, kinetic contribution of PFOS in suspended or bottom sediment to the body burden of fish, and the potential mechanisms responsible for this contribution. We also examined the proportional distribution of PFOS among different tissues of the fish.

## MATERIALS AND METHODS

### Experimental setup

The experiment was conducted with 88 2-yr-old marbled flounder (*Pseudopleuronectes yokohamae*, 22.2–90.4 g-wet; average 45.8 g-wet, standard deviation 13.7 g-wet on day 0 [just before the start of the exposure]) in flow-through polypropylene tanks (800 mm × 560 mm × 480 mm high), each holding approximately 160 L of seawater. The fish were obtained from a hatchery and reared at our institute for 21 mo. Three exposure treatments, in addition to a control treatment, were established to expose the fish to PFOS dissolved in water, associated with suspended sediment particles in water, or associated with bottom sediment. The control treatment (1 tank) received seawater with no added PFOS and contained no sediment. Water-exposure treatment (WAT, 2 tanks) received PFOS-spiked seawater and contained no sediment. Bottom-sediment-exposure treatment (BST, 2 tanks) received nonspiked seawater but contained a bed of sediment 1 cm to 2 cm thick that had been spiked with PFOS. The sediment bed was not renewed or added after the start of the experiment. Suspended-sediment-exposure treatment (SST, 1 tank) originally contained no sediment and received the effluent from one of the BST tanks. The bottom sediment in the BST was suspended by the activity of the fish. Both bottom and suspended sediments were therefore present in the BST. Water was well mixed in each tank of all treatments owing to aeration.

A 28-d exposure period was followed by an 84-d depuration period. The SST had only the exposure period. At the end of the exposure period, fish in the 2 tanks in each exposure treatment were combined and moved to a new tank, which contained no sediment and received nonspiked seawater during the depuration period. Fish mass loading was maintained at less than 2.5 (g-wet d)/L. The water temperature was kept at approximately 17.5 °C. We used seawater collected from a depth of 397 m in Suruga Bay, Japan, stored at our institute, and filtered through ceramic filters (nominal pore size 0.1 µm; NGK Insulators). A standard solution of the potassium salt of PFOS (100 mg/L) in methanol (PFOS-002S; AccuStandard) was diluted 5000-fold with Milli-Q water to make a PFOS stock solution, which was further diluted 200-fold with inflowing seawater. The nominal concentration of the PFOS anion in the spiked seawater was 93 ng/L. Fine sediment containing 2.5% (dry-mass basis) organic carbon was collected in March 2008 from a depth of approximately 10 m in Tokyo Bay, Japan, wet-sieved through 6-mm mesh to remove large materials, and stored at 4 °C. Four batches of sediment were then spiked with 2 mL each of the standard solution of the potassium salt of PFOS, hand-mixed with a scoop, left for 20 d at 4 °C, and placed in the BST tanks before the start of the experiment. The fish were fed commercial fish food at 0.5% of the average wet mass of the fish each day for 6 d per week. The feeding rate in each tank was adjusted for the sampled fish and by weighing all fish every 28 d. Unconsumed food, if present, and solid feces were regularly removed with a siphon, except during the exposure period of the sediment treatments because of turbidity.

### Sampling

Three flounders were sampled on day 0. Subsequently, 3 flounders were sampled from the control on days 28, 56, 84, and 112 and from the exposure treatments on days 3, 7, 14, and 28 (exposure period) and days 42, 56, 70, 84, and 112 (depuration period), the only exception being that 2 flounders were sampled on day 84 from the BST. An additional 2 and 3 flounders were sampled on day 28 from the WAT and BST, respectively, for tissue distribution determination. Multiple fish samples at each sampling represented and incorporated into the data analysis the variability between individual fish. One fish jumped out of a WAT tank and was excluded from the experiment. In the SST, 1 fish was found dead on day 28 and another fish was randomly excluded from the analysis.

Sampled flounders were washed with seawater, numbed in ice-cold seawater, measured and weighed, and then frozen (−20 °C). The samples were later thawed, and the body surface and gills were carefully washed to remove any remaining particles. For all of the 85 flounders that were analyzed, liver mass was recorded, and the gastrointestinal tract (gut) was opened, washed to remove any remaining sediment or food inside, and then included in the whole fish or viscera sample. From each of the 5 tissue-distribution samples, a blood sample was withdrawn immediately after weighing from the caudal vein with a glass syringe connected to a needle (23G × 1″; Terumo), both of which were wetted with a minimal volume of aqueous sodium heparin. The blood samples were stored in 1.5-mL polypropylene tubes and kept frozen until analysis. The 5 samples were dissected and separated into muscle, liver, gonad, other internal organs (viscera), and remainder (carcass). All fish samples were then kept frozen until analysis.

Water samples were taken on days 0, 1, 3, 5, 7, 10, 14, 18, 21, 24, 28, 29, 31, 42, 56, 70, 84, and 112 from the exposure treatments and on days 0, 1, 28, 56, 84, and 112 from the control treatment. The pairs of samples collected on days 10 and 14, 18 and 21, and 24 and 28 from the exposure treatments were composited. Sediment samples were taken immediately after emplacement of the sediment and on days 1, 14, and 28. The sediment interstitial water was sampled immediately after the sediment sampling by centrifuging an aliquot of the sediment samples at 1000 *g* for 20 min. Water and sediment samples were stored at 6 °C in the dark until analysis.

### Chemical analysis

The concentration of PFOS in the samples was determined according to previously reported methods with modifications (water and sediment 4, fish tissue 15, fish blood 3–16). The samples were spiked with ^13^C_4_-PFOS, and quantification was based on the isotope dilution method (see Supplemental Data, Sections S1–S2 for details). Dissolved-phase concentrations were measured for the interstitial water samples. Homogenized whole fish, fish tissues (other than sampled blood), and fish food samples were spiked with 5 ng of ^13^C_4_-PFOS, mixed with silica gel, and then extracted with 20% methanol (aqueous) using an accelerated solvent extractor. We used solid-phase cartridges (Presep-C Agri and Presep-C Alumina; Wako Pure Chemical Industries) to clean up the extracts. The cleaned-up eluate was finally concentrated to 1 mL in methanol. A 100-µL aliquot of the whole-blood samples was mixed with 1 mL of 0.5 M tetrabutylammonium solution, 2 mL of 0.25 M sodium carbonate buffer, and 5 ng of ^13^C_4_-PFOS in 10 µL of methanol. The mixture was extracted with methyl-tertiary-butyl ether by mixing and centrifugation. The extract was solvent-changed to 1 mL of acetonitrile and cleaned up by passage through a solid-phase cartridge (Oasis MCX; Waters). The cleaned-up eluate was finally solvent-changed to 0.2 mL of 90% methanol (aqueous) and filtered. Identification and quantitation were carried out by injecting an aliquot of the final concentrated extract into a liquid chromatograph connected to a triple-quadrupole tandem mass spectrometer equipped with a C18 liquid-chromatography column. The detection limit was 0.06 ng/mL and 0.02 ng/g-wet for blood samples 16 and whole-body or tissue samples, respectively. The detection limit of water samples was approximately 0.09 ng/L (dissolved phase) and approximately 0.03 ng/L (particulate phase). Values below the detection limit were treated as half the detection limit when necessary in the data analysis.

Lipid contents were determined gravimetrically using chloroform/methanol (2:1, v/v) as the extraction solvent. The concentrations of suspended solids in water samples were determined by weighing dried residue on a precombusted glass-fiber filter (nominal pore size 0.3 µm, GF-75; Advantec Toyo Kaisha). Dissolved organic carbon was measured as the nonpurgeable organic carbon in water sample filtrates using a total organic carbon analyzer. The organic carbon content in the sediment was measured with a CHN coder after removing inorganic carbonate. Normally, water temperature, pH, and dissolved oxygen (DO) concentrations were monitored every weekday and salinity and ammonia concentration (salicylate method) were montiored weekly. The particle-size distribution of the sediment was determined without pretreatment using a laser diffraction particle-size analyzer on the basis of equivalent sphere diameter and calculated in terms of volumetric percentages.

### Data quality assurance and quality control

Analytical reproducibility and method recovery of PFOS in water, sediment, and fish samples were confirmed to be satisfactory. No PFOS was detected in the method blank. See Supplemental Data, Section S3 for details.

### Respiration measurement

Oxygen-consumption rates of the fish were determined by using a separate set of marbled flounder (*n* = 6, average 31 g-wet). We kept individual fish in a sealed vessel filled with seawater for 1 h or 2 h and measured the decrease of oxygen in the water at 17.4 °C. The fish were calm during the measurement. Mass-specific oxygen consumption rate of the fish (*r* [1/T]) was calculated with Equation 1


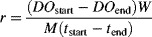
1

where *M* [M] is the fish's wet mass, *W* [V] is the measured volume of water in the vessel, *DO* [M/V] is the DO concentration, *t* [T] is time, and the subscripts “start” and “end” indicate the start and end of the measurement, respectively. The decrease in the DO concentration was corrected by the control (seawater only) measurement. The symbols M, V, and T in square brackets represent dimensions of mass, volume (length^3^), and time, respectively.

### Data analysis

To examine the statistical difference in the physiological parameters of 2 groups of fish, we used the Mann–Whitney rank sum test against the null hypothesis that the distributions of the 2 populations were the same. The median of the differences was estimated by the associated Hodges–Lehmann estimator 17. Statistical analyses were carried out with IBM SPSS Statistics (version 20; International Business Machines) and StatXact (version 8; Cytel).

*Kinetic model*. We analyzed the uptake and depuration by the fish based on a first-order kinetic of concentration (Equation 2) that was derived from a mass-balance of PFOS assuming fish as one compartment 8,18


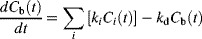
2

where *C*_b_ [M/M] is the concentration of PFOS in the fish as a function of time *t*, *i* indicates the exposure medium, *C*_*i*_ [M/M or M/V] is the concentration in the exposure medium, *k*_*i*_ [1/T or V/(M T)] is the rate constant for uptake from the exposure medium, and *k*_d_ [1/T] is the rate constant for collective depuration (elimination and metabolic transformation). The half-life equals ln(2)*k*_d_. In Equation 2, *C*_b_ needs to be appropriately corrected to account for dilution by the growth of fish. In the present study, *C*_*i*_(*t*) was approximated during the interval *t*_*k*_ ≤ *t* < *t*_*k*+1_ using the average of the 2 sample values at times *t*_*k*_ and *t*_*k*+1_. Integration of Equation 2 yields Equation 3 19. Equation 3 was applied in a consecutive manner to the time series of samples from each exposure medium.


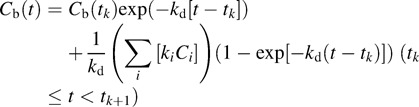
3

To assess the potential role of suspended and bottom sediments in the uptake of PFOS by fish, 3 models were employed (Equations 4–6) that differed in terms of the exposure medium being considered


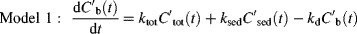
4


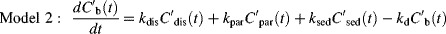
5


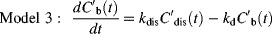
6

where the subscripts “dis,” “par,” “tot,” and “sed” indicate the dissolved phase, particulate phase, sum (total) of the concentrations in the dissolved and particulate phases in water, and sediment, respectively, and a prime symbol indicates subtraction of the control value. Both the water column and bottom sediment were considered as exposure media in models 1 and 2, whereas only the dissolved phase was considered in model 3. Model 1 used the total concentration in the water column, whereas the other models distinguished the dissolved phase from the particulate phase. The contribution of food to the PFOS body burden in fish, if any, was canceled out in all models by subtracting the concentrations in the control treatment.

#### Model fitting

The kinetic models were fitted by nonlinear fitting (CNLR command; SPSS), using the measured PFOS concentrations in fish and exposure media and assuming common rate constants among the treatments. The point estimate of the parameters (rate constants) was calculated based on least squares. The errors in the log-transformed data were assumed to have the same variance and to be uncorrelated (logOLS). To confirm the logOLS results, weighted least-squares fitting was also conducted by assuming errors to be uncorrelated and variances to be proportional to the squared model-predicted values. The 95% lower and upper bounds of the estimated parameters were calculated based on bootstrap resampling (*B* = 1000) of the concentrations in the fish (BOOTSTRAP subcommand; SPSS). Visual inspection of the overall fit and adjusted residual sum of squares (adjRSQ = RSQ/[*n* − 2*p*], where RSQ is the squared sum of the residuals, *n* is the number of data, and *p* is the number of parameters) 20 were used as the criteria for model selection. The adjRSQ takes into account the difference in the number of parameters between models. We also examined the results of fitting the models separately to each exposure treatment.

#### Uptake efficiency

An uptake rate constant (*k*_*i*_) can be expressed (Equation 7) as the product of the fish-mass-specific medium exposure rate (*e*_*i*_) [1/T or V/(M T)] and the corresponding uptake efficiency (*a*_PFOS, *i*_) [M/M] of PFOS from the exposure medium.


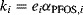
7

Regarding the uptake of PFOS from the respiratory surfaces, *e*_resp_ [V/(M T)] is the mass-specific ventilation rate and can be deduced from the mass-specific oxygen-consumption rate of the fish at the kinetic experiment (*r*_e_)


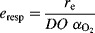
8

where 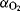 is the uptake efficiency of oxygen at the respiratory surfaces.

Equation 9 estimates *r*_e_ using the measured *r* (Equation 1)


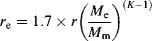
9

where *M*_m_ [M] is the mass of the fish at the respiration measurement, *M*_e_ [M] is the mass of the fish at the kinetic experiment, *K* is a parameter in an allometric relationship (Equation 10) to which we assigned a value of 0.78 21–22, and 1.7 is a correction factor to obtain routine metabolism 23.


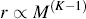
10

Substituting Equation 8 to Equation 7 yields the uptake efficiency of PFOS relative to that of oxygen (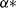) 24


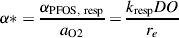
11

Regarding the uptake from the gut, *e*_sed_ [1/T] is the mass-specific ingestion rate of sediment and was conservatively assumed to equal the daily feeding rate of 0.5%. Equation 7 then gives the gut uptake efficiency of PFOS from sediment as


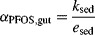
12

#### Tissue distribution

The amount of PFOS present in each tissue (or in blood) was calculated by multiplying the PFOS concentration by the wet mass of the tissue (or by blood volume). The blood volume was estimated by multiplying the wet mass of the fish by 35 mL/kg 25. In the present study, PFOS concentrations measured in tissues inevitably included a contribution from the blood present in the tissues. The amount of PFOS in each tissue was therefore not exclusive of the estimated amount of PFOS in the blood. The relative distribution of PFOS among tissues was examined against the sum of the amounts present in each tissue other than the amounts estimated in the blood.

## RESULTS

### Experimental conditions

The median diameter of the sediment particles was 23 µm. During the experiment, the water had an average ± standard deviation (all tanks) temperature of 17.3 ± 0.7 °C, pH 7.8 ± 0.1, DO concentration of 7.5 ± 0.5 mg/L (96% ± 6% saturation), NH_3_-N concentration of 0.22 ± 0.16 mg/L, and dissolved organic carbon concentration of 0.65 ± 0.19 mg C/L. Suspended-solid concentrations were 7.3 ± 1.3 mg/L, 9.3 ± 2.2 mg/L, 224 ± 171 mg/L, 8.2 ± 2.6 mg/L, and 174 ± 155 mg/L in the control, WAT, BST (exposure period), BST (depuration period), and SST, respectively.

Observable fish were active at the time of sampling from all treatments, with the exception of 1 dead fish in the SST, and at the time of feeding in the control and WAT (turbidity precluded observation of fish activity in the BST and SST). The median wet mass of the fish on day 0 was statistically significantly higher in the WAT than in the control (median of difference 10.3 g, 95% confidence interval 2.3–19.5 g). No adjustment was undertaken because the fish had been randomly assigned to the treatments. There were no statistically significant differences in the median wet mass of the fish in the BST or SST versus the control. Because the wet mass of the fish showed no statistically significant trend (either linear or exponential) with time in any treatment, no growth correction was applied in the kinetic analysis. The lipid content of the fish and the hepatosomatic index (liver mass/body mass) had a median of 3.0% (*n* = 80) and 1.3% (*n* = 85), respectively, and showed in any treatment no statistically significant trend with time nor statistically significant difference from the control.

### PFOS concentration

**Figure 1:**
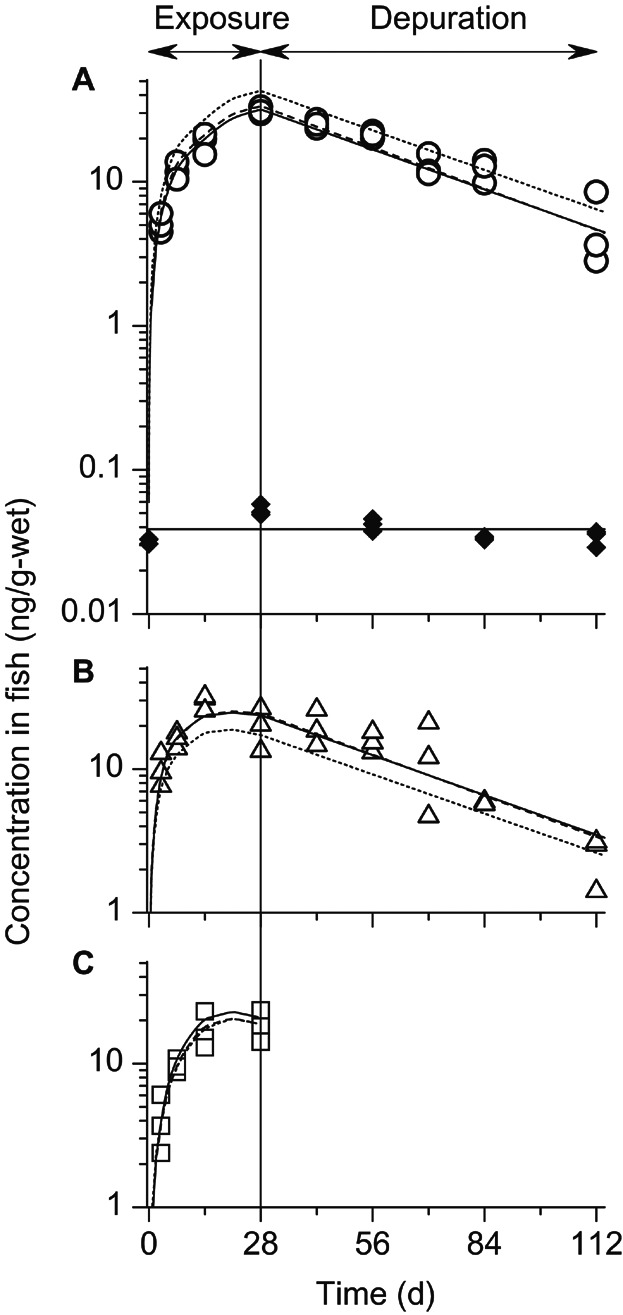
Time course of PFOS concentrations in fish and model prediction curves based on kinetic models. Treatments: (A) control and day 0 (diamonds) and water exposure (circles); (B) bottom-sediment exposure (triangles); and (C) suspended-sediment exposure (squares, no depuration period). Solid, dashed, and dotted curves for exposure treatments show models 1, 2, and 3, respectively, fitted to the data. The horizontal line in (A) shows the average of day-0 and control samples.

The PFOS concentrations in the fish are shown in Figure 1, and the PFOS concentrations in the water, sediment, and sediment interstitial water samples are shown in Supplemental Data, Figure S1. Dissolved and particulate PFOS concentrations during the exposure period in the WAT were relatively constant and averaged 74 ng/L and 18 ng/L, respectively. Dissolved and particulate concentrations in the BST peaked on day 1 at 90 ng/L and on day 3 at 100 ng/L, respectively. These concentrations increased 5-fold after fish were added to the BST tanks at the start of the experiment and the sediment was suspended by fish activity. Both dissolved and particulate PFOS concentrations decreased afterward. Those in the SST peaked on day 3 at about 50 ng/L to 60 ng/L, were relatively constant up to day 14, and then decreased. The PFOS concentration in the BST sediment decreased from 110 ng/g-dry (on emplacement, coefficient of variation = 13%, *n* = 8) to 15 ng/g-dry (day 28). The PFOS concentrations were negligible in the water in the control, in the exposure treatments during the depuration period (average WAT, 0.66 ng/L; BST, 0.37 ng/L), and in the control fish (average 0.04 ng/g-wet). In the food, PFOS was not detected (<0.15 ng/g).

### Kinetic analysis

The estimated rate constants are shown in Table1. The estimates by the alternative weighted least-squares fitting were comparable to those using logOLS. The curves predicted by models 1 and 2 were similar and were almost identical for the WAT and BST (Figure 1). Model 3 apparently overpredicted and underpredicted PFOS concentrations in the WAT and BST, respectively. The adjRSQ values for models 1 and 2 were comparable and approximately 40% better than the adjRSQ value for model 3 (Table1).

**Table 1:** Rate constants of perfluorooctane sulfonate for whole body of marbled flounder estimated by kinetic models, corresponding uptake efficiency or half-life, and a measure of fit of each model

Model	adjRSQ^a^	Rate constant^b^^c^		Uptake efficiency^b^	Half-life (d)^b^
1	0.099	*k*_tot_	18 (17–20)	0.027 (0.024–0.030)^d^	
		*k*_sed_	5.0 (1.2–9.0)	4.0 (0.85–7.1)^e^	
		*k*_d_	0.023 (0.020–0.026)		30 (27–35)
2	0.099	*k*_dis_	22 (18–26)	0.032 (0.026–0.038)^d^	
		*k*_par_	11 (3.6–20)	0.016 (0.0039–0.028)^d^	
		*k*_sed_	7.1 (2.1–12)	5.7 (1.9–9.5)^e^	
		*k*_d_	0.024 (0.020–0.027)		29 (26–34)
3	0.16	*k*_dis_	31 (27–35)	0.046 (0.040–0.052)^d^	
		*k*_d_	0.023 (0.019–0.026)		31 (27–36)

a Adjusted residual sum of squares, as a measure of fit. adjRSQ = RSQ/(*n* − 2*p*), where RSQ is the squared sum of the residuals, *n* is the number of data, and *p* is the number of parameters.

b Values are presented as point estimate (95% confidence interval).

c Units of the rate constant are L/(kg-wet-fish d) for *k*_tot_, *k*_dis_, and *k*_par_; g-dry-sed/(kg-wet-fish d) for *k*_sed_; and 1/d for *k*_d_.

d Uptake efficiency relative to that of oxygen, assuming respiratory uptake.

e Gut uptake efficiency, assuming sediment ingestion rate equal to that of food.

*k* = rate constant; tot = sum (total) of the concentrations in the dissolved and particulate phases in water; sed = sediment; d = depuration; dis = dissolved phase; par = particulate phase

Treatment-specific fitting of model 1 resulted in *k*_tot_ values of 18 L/(kg-wet-fish d), 17 L/(kg-wet-fish d), and 17 L/(kg-wet-fish d) for the WAT, BST, and SST, respectively, and a *k*_sed_ value of 10 g-dry-sed/(kg-wet-fish d) for the BST. Model 2 did not yield statistically significant uptake parameters, probably because of the correlation between the PFOS concentrations in the dissolved and particulate phases within each treatment. Model 3 resulted in *k*_dis_ values of 22 L/(kg-wet-fish d), 47 L/(kg-wet-fish d), and 30 L/(kg-wet-fish d) for the WAT, BST, and SST, respectively.

Under the experimental conditions, the contributions from each exposure medium to the PFOS concentration in fish at day 28 as predicted by the kinetic models (Supplemental Data, Figure S2) were 77% from *C*_tot_ and 23% from *C*_sed_ in the BST based on model 1; 49% from *C*_dis_, 19% from *C*_par_, and 32% from *C*_sed_ in the BST based on model 2; and 70% from *C*_dis_ and 30% from *C*_par_ in the SST based on model 2, respectively.

### Tissue distribution

In both the WAT and BST, PFOS concentrations in tissues were found to decline from high to low in roughly the order blood, gonad, liver, viscera, carcass, and muscle. The PFOS concentrations ranged from 8.8 to 100 and 8.1 to 180 ng/g wet (tissue) or ng/mL (blood) among tissues in the WAT and BST, respectively (Table2). A major part (37–66%) of the mass of PFOS in fish was present in the carcass (Supplemental Data, Figure S3). The PFOS in the gonad constituted >25% of the PFOS in female fish and <11% in male or gender-unidentified fish. Liver and viscera (excluding liver and gonad) each contributed less than 5% to the mass of PFOS in fish.

**Table 2:** Concentrations of perfluorooctane sulfonate (PFOS) in fish tissues (ng/g) and in whole blood (ng/mL) and body mass (g-wet) and gender of each fish

PFOS concentration								
Sample	Body mass	Gender	Blood	Gonad	Liver	Viscera^a^	Carcass	Muscle
WAT	72.1	F	100	64	54	55	28	9.5
WAT	61.0	F	85	78	67	46	27	8.8
BST	76.2	M	140	180	91	77	32	12
BST	63.8	F	72	57	70	48	22	8.1
BST	37.6	—b	130	73	90	80	36	15

a Viscera do not include gonad and liver.

b Gonad too small to judge male or female.

F = female; M = male (judged by gonad observation); WAT = water-exposure treatment; BST = bottom-sediment-exposure treatment.

## DISCUSSION

### Kinetic analysis

We adopted a general form of integration (Equation 3) of the kinetic equation to account for changes in the PFOS concentrations in the exposure media, which we anticipated, particularly in the BST and SST. The kinetic models successfully represented the observed PFOS concentrations in fish, including the decrease in the BST after day 14 and the plateau at the same time in the SST, patterns that reflected the decrease in the PFOS concentrations in the water and sediment (Supplemental Data, Figure S1). The PFOS contained in the food had a negligible impact on the PFOS concentration in fish, as demonstrated by the low measured concentrations in the control fish and no apparent increase thereof with time (Figure 1A).

### Comparison with previously reported rate constants and uptake efficiencies

We consider the value of the uptake rate constant from the dissolved phase (*k*_dis_) obtained from model 2 to be most representative of the respiratory uptake of PFOS among the *k*_dis_ values obtained in the present study. This interpretation was supported by the identical *k*_dis_ value obtained from model 3 applied only to the results from the WAT.

The values obtained for the kinetic parameters (Table1) were comparable to those previously reported for PFOS in other fish species. Both the *k*_dis_ (22 L/[kg-wet-fish d]) obtained from model 2 and the *k*_d_ (0.023–0.024 [1/d], corresponding to a half-life of 29–31 d) estimated by model 1 to model 3 lay in the range reported for fish whole bodies or carcasses of carp (*Cyprinus carpio*) 26, bluegill (*Lepomis macrochirus*) 27, and rainbow trout (*Oncorhynchus mykiss*) 7–28 (*k*_dis_, 10–53 L/[kg-wet-fish d]; *k*_d_, 0.0062–0.054 [1/d], corresponding to a half-life of 13 d–110 d). The bioconcentration factor for PFOS, calculated as the ratio of model-2 *k*_dis_ and *k*_d_ values, was 920, which was similar to the bioconcentration factor values reported for fish whole bodies or carcasses of other fish species (720 26, 2800 27, and 1100 7).

Comparison of the estimated rate constants with those previously reported for other surfactants supported the observation that metabolic transformation of PFOS may be negligible 7 and suggested that the respiratory uptake of PFOS shares a common mechanism with other surfactants. Chemical uptake and depuration kinetics in fish have been related to the phase distribution of chemicals between water and organic matter representing the fish body 19. We here adopt the critical micelle concentration (CMC) as a measure of the phase distribution of surfactants 29. In aqueous solution, surfactants form aggregates, or micelles, above a certain concentration, that is, the CMC 29. The more hydrophobic a surfactant is, the less thermodynamically favorable it is for the surfactant molecules to remain dissolved in the water; thus, micelles form at a lower concentration 29. The octanol–water partition coefficient is a common measure of the phase distribution for nonionic chemicals, but its direct measurement is problematic for surfactants including PFOS 7. The *k*_d_ values of surfactants have been reported to range from 0.12 to 2.2 (1/d) for log CMC values (mol/L) in the range of −0.5 to −5.5 29. Log CMC values of major salts of PFOS have been reported to be approximately −2.1 30, which is within this range. But the *k*_d_ value obtained in the present study was low compared to the *k*_d_ values of these other surfactants, suggesting a slower metabolic transformation of PFOS compared to the other surfactants. In contrast, the linear relationship between the *k*_dis_ and CMC of surfactants 29 predicted the *k*_dis_ of PFOS to be 12 L/(kg-wet-fish d), which was close to our estimated value.

The obtained respiratory uptake efficiency of PFOS was comparable to efficiencies estimated for PFOS in other fish species and was lower than those typically reported for neutral hydrophobic compounds in fish. Uptake efficiency provides a better comparison of uptake kinetics between compounds or species because the uptake rate constant depends on the ventilation rate (Equation 7), which depends on the size and species of fish. There are few reported respiratory uptake efficiencies for PFOS 31. Ankley et al. 31 analyzed rainbow trout data 7 with the use of Equation 11, published respiration data, and suggested a respiratory uptake efficiency in juvenile rainbow trout of approximately 0.095 that of oxygen (i.e., 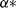). We likewise analyzed the PFOS *k*_dis_ in carp 26 and bluegill 27 with Equations 9 and 11 as well as literature respiration data 32–33. The analysis yielded an estimate for 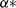 of approximately 0.007 to 0.02. These 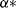 values were all comparable in magnitude to our result (0.032). Factors such as water type (freshwater or seawater), fish species, exposure concentration, and differences in mode of exposure (single compound or mixture) may account for the differences. The respiratory uptake efficiency of PFOS was lower than those typically reported for neutral hydrophobic compounds (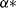 > 0.3 for log [octanol–water partition coefficient] > 1.5), although somewhat lower values have been found for more hydrophilic compounds (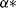, 0.11–0.14) and for some polycyclic aromatic hydrocarbons (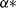 < 0.3) 6 (J. Kobayashi et al., National Institute for Environmental Studies, Tsukuba, Japan, unpublished data). No reported values were available for 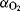 for marbled flounder, which is necessary to calculate 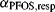. If the 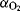 value of 0.53 for Japanese flounder (*Paralichthys olivaceus*) 34 is used, 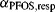 would be 0.017.

### Tissue distribution

The observed distribution of PFOS among tissues was comparable to that reported for rainbow trout in the laboratory 7–35 and for European chub in the field 36 in that higher concentrations were observed in the blood, liver, and gonad. Comparable PFOS levels in the liver and gonad were also observed in laboratory-exposed fathead minnow 37. The authors of these studies suggested that binding to protein, high blood perfusion of an organ, or both contribute to the observed levels in these organs 7–37.

The reported distribution of polychlorinated biphenyls (PCBs) in the tissues of 1-yr-old marbled flounder (14% muscle, 7.2% liver, 1.8% internal organs including gonad, and 77% carcass) 11 was generally similar to that observed in the present study. The smaller proportion of PCBs in the gonad may have resulted from immaturity of the 1-yr-old fish.

### Potential uptake of PFOS from particulate phase and bottom sediment

The results suggested that the PFOS in suspended or bottom sediment contributed to the observed body burden in the fish. The better fit of models 1 and 2 demonstrated that these models were most suitable to interpreting the experimental results and that the PFOS in suspended and bottom sediments contributed to the observed body burden in fish. The values obtained for the uptake rate constant from these media were not trivial (i.e., *k*_par_ or *k*_tot_ of the same order of magnitude as *k*_dis_ and *k*_sed_ corresponding to 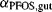 > 100%; see also Supplemental Data, Figure S2) and were statistically significant (Table1). It was difficult to explain the higher *k*_dis_ values estimated from the sediment treatments by applying model 3 to each treatment. In contrast, consistent uptake parameters obtained by applying model 1 to each treatment suggested that a model incorporating the particulate phase and bottom sediment as exposure media was valid. Overestimating and underestimating PFOS concentration in fish in the WAT and BST, respectively, by model 3 suggested that the body burden of PFOS was not fully accounted for by the measured PFOS concentration in the dissolved phase.

Several factors (including physiological mechanisms) not specifically considered in our calculations may have contributed to the observed uptake rate constants of PFOS from suspended and bottom sediments. We used observed data and information in the literature (Table3) to evaluate the possible contribution of these factors, which included sediment particles remaining in fish, changes in DO and ventilation rate, uptake of PFOS from ingested sediment particles, water drinking, and cutaneous uptake.

**Table 3:** Potential factors contributing to observed uptake rate constant from particulate phase and bottom sediment

Factors	Treatment	Mechanism	Likely contribution (order of magnitude estimate)
Sediment particles remaining in fish	BST, SST	Experimental artifact	Up to 3%–8% of *k*_tot_ or *k*_par_ (0.5–0.9 L/[kg-wet-fish d]) or up to 6%–8% of *k*_sed_ (0.4 g-dry-sed/[kg-wet-fish d])
DO and ventilation rate	BST, SST	Depleted DO leading to increased ventilation rate and thus increased PFOS uptake	Unlikely because of minimal difference in water-column DO among treatments
Ingestion of sediment particles	BST (also possible in SST)	PFOS sorbed to sediment particles concomitantly ingested is taken up in the gut	25% at maximum, likely up to 2.5% of *k*_sed_
Water drinking	BST, SST	Marine fish ingest seawater for osmotic regulation, and suspended sediment particles may enter the gut with the water; the associated PFOS may be taken up through the intestinal epithelium and thus contribute to *k*_par_	Up to 0.4 L/(kg-wet-fish d)
Cutaneous respiration	BST	Cutaneous uptake of PFOS in sediment interstitial water	Roughly 10% of *k*_sed_

BST = bottom-sediment-exposure treatment; SST = suspended-sediment-exposure treatment; DO = dissolved oxygen; PFOS = perfluorooctane sulfonate; *k* = rate constant; tot = sum (total) of the concentrations in the dissolved and particulate phases in water; par = particulate phase; sed = sediment.

#### Sediment particles remaining in fish

The mass of sediment particles remaining in the fish samples would be minimal, no more than roughly 0.1 g-wet, because we carefully washed and removed sediment particles from the body surface, gill, and inside of the gut. This amount of sediment would contribute only 0.16% to 0.82% (BST) or 0.87% to 3.0% (SST) of the PFOS in the whole fish samples and would result in an uptake rate constant of approximately 0.5 (BST) or 0.9 (SST) L/(kg-wet-fish d), which was equivalent to a *k*_sed_ of approximately 0.4 g-dry-sed/(kg-wet-fish d).

#### DO and ventilation rate

The assumption that the rate constants were common among the treatments would have resulted in the poorer fit of model 3 if the levels of DO differed among treatments because a low DO would increase ventilation rates (Equation 8) and thereby change uptake efficiency and resulting *k*_dis_. However, because the measured DO in the water column varied little among treatments (average during the exposure period: WAT, 7.5 mg/L; BST, 7.3 mg/L; SST, 7.5 mg/L) and within tanks, this factor likely contributed little.

#### Ingestion of sediment particles

The estimated gut uptake efficiency from ingested sediment particles of >400% (Table1) suggested that this uptake mechanism could account for at most 25% (=100%/400%) of the observed *k*_sed_ because an uptake efficiency >100% is unreasonable. This contribution (25%), however, was probably an overestimate. The assumption that the sediment ingestion rate equaled the feeding rate was probably an overestimate because only a small amount of sediment particles was observed in the gut and because the fish are able to flush unwanted materials through gill clefts after ingestion. Assuming a sediment ingestion rate of 10% of the feeding rate would result in uptake from ingested sediment particles accounting for <2.5% of the observed *k*_sed_.

#### Water drinking

Marine fish ingest seawater for osmotic regulation 38, and the PFOS associated with suspended sediment in the water may be taken up from the gut as PFOS in food and thus contribute to *k*_par_. However, Tulp et al. 38 have already concluded that the contribution of dissolved pentachlorobiphenyl in ingested water is negligibly small for salmon compared with uptake from the respiratory surfaces. A similar calculation based on Equation 7, using water-drinking rates from the literature 38–39 and an assumed gut uptake efficiency of 100%, revealed a negligible contribution from the particulate-phase PFOS in ingested water for marbled flounder (up to 0.4 L/[kg-wet-fish d]).

#### Cutaneous uptake

In addition to the gills, the body surface in general contributes to gas exchange 40 and may contribute to uptake of PFOS as well. Marbled flounder often rest with their body submerged in bottom sediment, with only their heads and gills out above the sediment. By assuming PFOS uptake to be proportional to the product of surface area and the inverse of the thickness of the epithelium or epidermis and adopting literature data 40 for these values, cutaneous uptake in the flounder was estimated to account for roughly one-three hundredth of the uptake via the gills. Taking into account the measured different PFOS concentrations in the water column and sediment interstitial water, this calculation led to a potential cutaneous uptake of PFOS from sediment interstitial water that equaled roughly 3% of the uptake from the water column and accounted for approximately 10% of the observed *k*_sed_.

All of the factors and physiological mechanisms that we examined played a minor role in the observed rate constants of the uptake of PFOS from suspended and bottom sediments (Table3), and other potential mechanisms responsible for the uptake need to be investigated. Kobayashi et al. 11 likewise reported that an average of 44% of the PCB body burden in marbled flounder was not explained by the measured dissolved PCB concentrations, in a similar exposure experiment with sediment in a semistatic system. In the BST and SST, the partitioning of PFOS in the water column was below equilibrium in the dissolved phase (compared with that in the spiked sediment); thus, PFOS would have been supplied via diffusion from the bottom sediment and particulate phase. This supply was already accounted for by the *C*_dis_ (or *C*_tot_) term in the kinetic models. The observed kinetic uptake from the suspended and bottom sediments would therefore result from a mechanism that was not associated with respiratory uptake from the dissolved phase or one that was local and not reflected in the water column *C*_dis_.

## SUPPLEMENTAL DATA

**Sections S1–S3. Figures S1–S3.** (190 KB PDF).

## Supporting Information

All Supplemental Data may be found in the online version of this article.

Acknowledgment—J. Nakano of the National Institute for Environmental Studies helped in the analysis of dissolved organic carbon
