# Supplementary material for: Transfer kinetics of perfluorooctane sulfonate from water and sediment to a marine benthic fish, the marbled flounder (Pseudopleuronectes yokohamae)
Source: Environ Toxicol Chem. 2013 Jul 11;32(9):2009–17. doi: 10.1002/etc.2270 (PMC3881517; doi:10.1002/etc.2270)
Supplement: Supplementary file 1 — Acknowledgment—J. Nakano of the National Institute for Environmental Studies helped in the analysis of dissolved organic carbon [file etc0032-2009-sd1.pdf]

Supplemental Data

Journal

Environmental Toxicology and Chemistry

Article Title

Transfer kinetics of perfluorooctane sulfonate (PFOS) from water and sediment to a marine benthic fish, the marbled flounder (*Pseudopleuronectes yokohamae*)

Authors

Takeo Sakurai *[†], Jun Kobayashi [†‡], Kyoko Kinoshita [†], Nozomi Ito [†], Shigeko Serizawa [†], Hiroaki Shiraishi [†], Jeong-Hoon Lee [†§], Toshihiro Horiguchi [†], Hideaki Maki [†], Kaoruko Mizukawa [†||#], Yoshitaka Imaizumi [†], Toru Kawai [†], Noriyuki Suzuki [†]

[†] National Institute for Environmental Studies, Onogawa 16–2, Tsukuba, Ibaraki 305–8506, Japan

[‡] Faculty of Environmental & Symbiotic Sciences, Prefectural University of Kumamoto, Tsukide 3–1–100, Kumamoto, Kumamoto 862–8502, Japan

[§] Southeast Sea Fisheries Research Institute, National Fisheries Research and Development Institute, Tongyeong, Gyeongnam 650-943, Korea

[||] Institute of Symbiotic Science and Technology, Tokyo University of Agriculture and Technology, Saiwaicho 3–5–8, Fuchu, Tokyo 183–8509, Japan

[#] Research Fellow of the Japan Society for the Promotion of Science, 8 Ichibancho, Chiyoda, Tokyo 102–8472, Japan

*Corresponding author, tsakurai@nies.go.jp

This supplementary contains 12 pages, including 3 figures.

S1 Standards and reagents

A methanol solution (100 mg/L, PFOS-002S) of potassium heptadecafluorooctanesulfonate (K-PFOS) was obtained from AccuStandard (New Haven, CT, USA), and used for both exposure and analysis. $^{13}C_4$-labeled sodium PFOS (98%, 1-[1,2,3,4-$^{13}C_4$]) in methanol was purchased from Wellington Laboratories (Guelph, ON, Canada). Pesticide- or HPLC-grade methanol and HPLC-grade acetonitrile were purchased from Wako Pure Chemical Industries, Ltd. (Osaka, Japan), Kanto Chemical Co., Inc. (Tokyo, Japan), or Nacalai Tesque, Inc. (Kyoto, Japan). Pesticide-grade methyl-tertiary-butyl ether (MTBE), special-grade tetrabutylammonium (TBA) hydrogensulfate, special-grade ammonium acetate, and solid-phase-extraction (SPE) cartridges Presep-C Agri (Short) (styrene-divinylbenzene–metacrylate polymer, 200 mg), and Presep-C Alumina (1700 mg) were obtained from Wako Pure Chemical Industries. An SPE cartridge BondElut C18 (500 mg, 10 mL) was obtained from Varian (Palo Alto, CA, USA), and OASIS MCX (3 cc, 60 mg) from Waters (Milford, MA, USA). Silica gel (Silica gel 60) was obtained from Kanto Chemical. Sodium heparin was obtained from Nacalai Tesque, Inc. (Kyoto, Japan). Milli-Q water was used in the chemical analyses.

S2 Chemical analysis

The chemical analysis of PFOS was performed according to previously reported methods with modifications (water and sediment, [S-1]; fish tissue, [S-2]; fish blood, [S-3, S-4]). The samples were spiked with $^{13}C_4$-PFOS, and quantification was based on the isotope dilution method.

Water samples were separated into dissolved and particulate phases by filtration through glass-fiber filters (nominal pore size, 0.3 μm, GF-75; Advantec Toyo Kaisha, Ltd., Tokyo, Japan). The dissolved phase (filtrate) was extracted by using SPE (C18). The particulate phase (residue on filter) and dried sediment samples were extracted by sonication in methanol, and the extract was diluted and purified by using SPE (C18) [S-1]. Dissolved phase concentrations were measured for the interstitial water samples.

Whole fish, fish tissues (other than blood), and fish food were homogenized (8000 rpm, 5 min; BLAS-501, Nihonseiki Kaisha Ltd., Tokyo, Japan). A 5-g aliquot (whole fish, muscle, and carcass) of the homogenate or all homogenate (other tissues) was taken, spiked with 5 ng of $^{13}C_4$-PFOS in 10 μL methanol, and mixed with silica gel. The mixed homogenate was extracted three times with 20% methanol (aq) by using an accelerated solvent extractor (ASE-200, Dionex, Sunnyvale, CA, USA), at 100 °C and 10 MPa. The extracts were combined, diluted with water to a methanol concentration <10%, and then passed through Presep-C Agri connected after Presep-C Alumina. The cartridges were washed with 10 mL of water, Presep-C Alumina was removed, and Presep-C Agri was dried and then eluted with 2 mL of methanol. The eluate was concentrated to 1 mL under a stream of nitrogen gas.

A 100 μL aliquot of the whole blood samples was mixed with 1 mL of 0.5 M TBA solution, 2 mL of 0.25 M sodium carbonate buffer, and 5 ng of $^{13}C_4$-PFOS in 10 μL methanol. The mixed solution was extracted by mixing with 5 mL of MTBE for 1 min, and the MTBE layer was taken after centrifugation (1400×*g*, 10 min; model 5100, Kubota Manufacturing, Gunma, Japan). The remaining aqueous layer was extracted with MTBE again. The two MTBE fractions were combined, dried under a stream of nitrogen gas, dissolved in 1 mL of acetonitrile, passed through an Oasis MCX cartridge, eluted with 10 mL of acetonitrile, dried under a stream of nitrogen gas, dissolved in 0.2 mL of 90% methanol (aq), and filtered (Autovial 5, 0.2 μm, PTFE, Whatman, Maidstone, Kent, UK).

Identification and quantitation were conducted by injecting 5 μL (biological samples) or 10 or 5 μL (abiotic samples) of the final concentrated extract into a liquid chromatograph connected to a triple-quadrupole type tandem mass spectrometer (LC–MS/MS) (biological samples: LC, HP1100 [Agilent Technologies, Santa Clara, CA, USA]; MS/MS, API4000Q TRAP [Applied Biosystems, Life Technologies, Carlsbad, CA, USA]; abiotic samples: HP1200–Agilent 6460 [Agilent Technologies]). An Xterra MS C18 LC column (2.1 mm i.d., 150 mm long, 3.5 μm particle size, Waters) was used for separation at a flow rate of 0.2 mL/min of a binary solvent system of acetonitrile and 10 mmol/L ammonium acetate in water as a mobile phase; starting at 45% acetonitrile for 4 min, ramped linearly to 90% at 10 min, kept at 80% for 3 min, and then dropped to 45%. For the water samples collected on days 84 and 112, a Zorbax Eclipse Plus C18 LC column (2.1 mm i.d., 100 mm long, 1.8 μm particle size, Agilent Technologies) was used for separation at a flow rate of 0.4 mL/min of the mobile phase; starting at 20% acetonitrile for 0.2 min, ramped linearly to 90% at 5 min, kept at 90% for 1 min, and then dropped to 20%. The MS/MS was operated with electrospray ionization (negative) in multiple reaction

monitoring mode. The capillary voltage, precursor ion, product ion, cone (or fragmentor) voltage, and collision energy were respectively as follows: API4000Q TRAP, 4.5 kV, m/z 499 ($[M-H]^-$), m/z 80 ($[SO_3]^-$), 105 V, 92 eV; Agilent 6460, 3.5 kV, m/z 499 ($[M-H]^-$), m/z 99 ($[FSO_3]^-$), 160 V, 45 eV. Quantitation was based on the isotope dilution method. The detection limit was 0.02 ng/g-wet for whole body or tissue samples, determined as three times the standard deviation of seven replicate analyses. The detection limit of the blood sample was previously established as 0.06 ng/mL [S-4] similarly based on seven replicate analyses. The detection limit for each water sample was determined as corresponding to a signal-to-noise ratio of 8, and ranged from 0.02 to 0.4 (median, 0.09) ng/L (dissolved phase) and from 0.01 to 0.5 (median, 0.03) ng/L (particulate phase) in the non-detect samples. Values below the detection limit were treated as half the detection limit when necessary in the data analysis.

Lipid contents were determined gravimetrically using chloroform/methanol (2:1) as the extraction solvent [S-5]. Homogenized samples (5 g) were dehydrated by mixing with anhydrous sodium sulfate and Soxhlet-extracted for 8 h with chloroform/methanol (2:1). The extract was concentrated to 1 to 2 mL and dissolved in 25 mL of petroleum ether. A 10 mL aliquot of the supernatant was taken, the solvent was evaporated, and the residue was dried at 100 °C for 1 h. The lipid content was determined by weighing the residue.

For suspended solids (SS) and dissolved organic carbon (DOC) measurements, the water samples were filtered through pre-combusted glass-fiber filters (GF-75). The residue on the filter was dried at 80 °C overnight, and the mass concentration of SS was obtained gravimetrically. The DOC concentration in the filtrate was measured as the non-purgeable organic carbon by using a total organic carbon analyzer (TOC-5000, Shimadzu Corp., Kyoto,

Japan) by combustion/non-dispersive infrared gas analysis, after acidification followed by bubbling with nitrogen gas. The organic carbon content in the sediment was measured with a CHN coder (Flash EA 1112, Thermo Electron Co., Waltham, MA, USA) after pretreatment of ground and dried (105 °C × 2 h) sediment with HCl (aq) to remove carbonates.

Normally, water temperature, pH (MH-20P, DKK-TOA Corp., Tokyo, Japan), and dissolved oxygen (DO) concentrations (LDO, Hach, Loveland, CO, USA) were monitored on every weekday, and salinity (S/Mill-E, Atago Co., Ltd, Tokyo, Japan) and ammonia concentration (salicylate method, DR4000, Hach) weekly.

The particle-size distribution of the sediment was determined without pretreatment by using a laser diffraction particle-size analyzer (SALD-2100, Shimadzu, Kyoto, Japan) on the basis of equivalent sphere diameter and was calculated in terms of volumetric percentages.

S3 Data quality assurance and quality control

Refer to Sakurai et al. [S-1] for data quality assurance and quality control of the analysis of water and sediment samples. Analytical reproducibility of PFOS in the whole fish and fish tissue samples was determined by means of seven replicate analyses of pooled unspiked 5-g samples of fish muscle. The average concentration and CV were 57 ng/g-wet and 8.4%, respectively. Method recovery was checked by seven replicate analyses of pooled muscle samples spiked at a nominal concentration of 0.80 ng/g. The recovery was satisfactory, 98.4% (SD, 4.2%). PFOS was not detected in the method blank samples (fish and fish tissue, n = 7; blood, n = 3). Analytical reproducibility and method recovery of blood samples were previously established as follows [S-4]. Based on seven replicate analyses of pooled unspiked 0.5 mL samples of human whole blood, the average concentration and CV were 1.0 ng/mL and 2.0%, respectively. Method recovery was satisfactory, 95.6% (SD, 3.3%), based on seven replicate analyses of pooled blood samples spiked with a nominal concentration of 8.0 ng/mL of PFOS.

References

S-1. Sakurai T, Serizawa S, Isobe T, Kobayashi J, Kodama K, Kume G, Lee J-H, Maki H, Imaizumi Y, Suzuki N, Horiguchi T, Morita M, Shiraishi H. 2010. Spatial, phase, and temporal distributions of perfluorooctane sulfonate (PFOS) and perfluorooctanoate (PFOA) in Tokyo Bay, Japan. *Environ Sci Technol* 44:4110–4115.

S-2. Japan's Ministry of the Environment. 2004. Chemicals in the Environment — Report of Development of Analytical Methods for Environmental Surveys. Tokyo, Japan. (in Japanese)

S-3. Hansen KJ, Clemen LA, Ellefson ME, Johnson HO. 2001. Compound-specific, quantitative characterization of organic fluorochemicals in biological matrices. *Environ Sci Technol* 35:766–770.

S-4. Japan's Ministry of the Environment. 2010. Report of the FY2009 Survey on the Accumulation of Dioxins and Other Chemical Compounds in Humans. Tokyo, Japan. (in Japanese)

S-5. Pharmaceutical Society of Japan. *Methods of Analysis in Health Science*, 2010 ed. Kanehara & Co., Ltd., Tokyo, Japan.. (in Japanese)

Figures

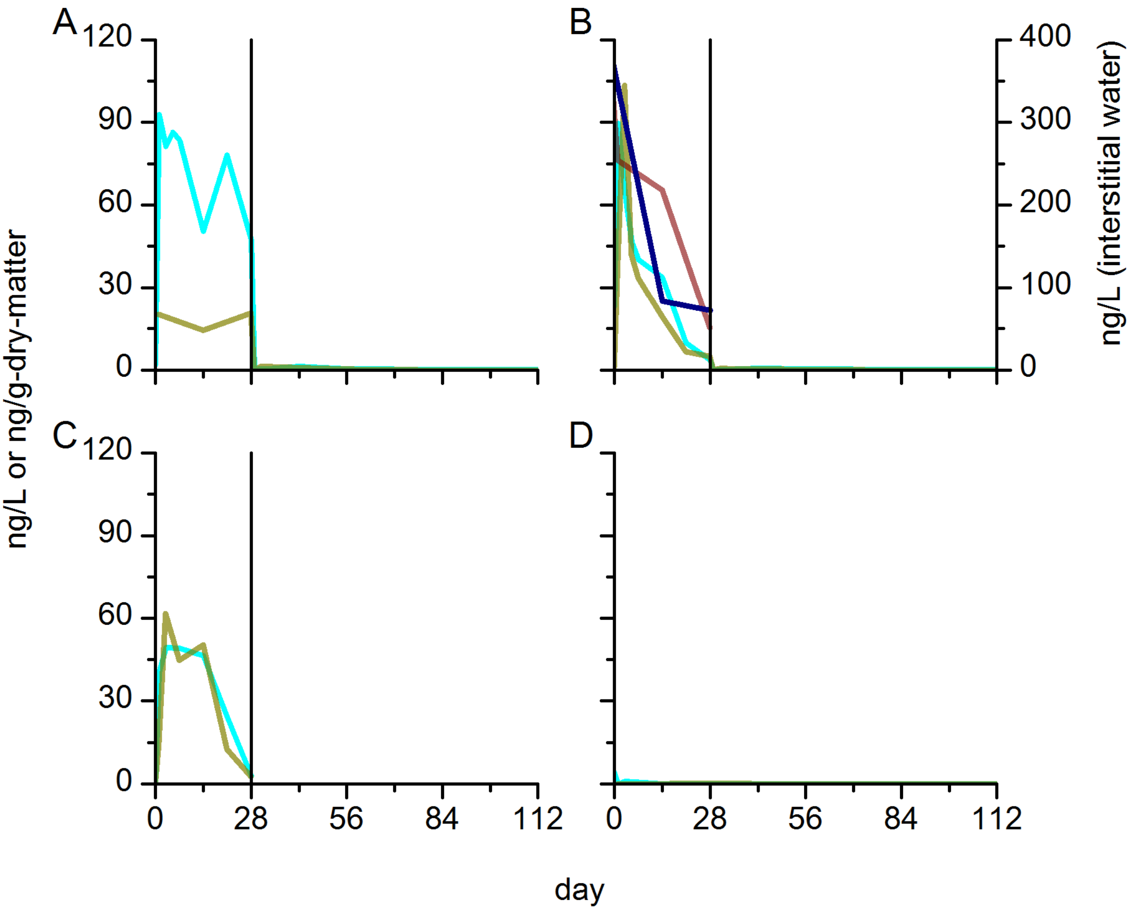

Figure S1. PFOS concentration in exposure media. (A) Water-exposure treatment (WAT); (B) bottom-sediment-exposure treatment (BST); (C) suspended-sediment-exposure treatment (SST); and (D) control. Blue line shows the concentration in the dissolved phase (ng/L), yellow the particulate phase (ng/L), brown the bottom sediment (ng/g-dry), and dark blue the sediment interstitial water (ng/L, right axis in Panel B). Exposure period was day 0 to day 28 in the three exposure treatments. There was no depuration period for the SST. Values below the detection limit were plotted as half the detection limit. The values in the samples composited on two different days are plotted on the later day of the samplings. Note that the concentration in bottom sediment upon emplacement was not shown in the plot.

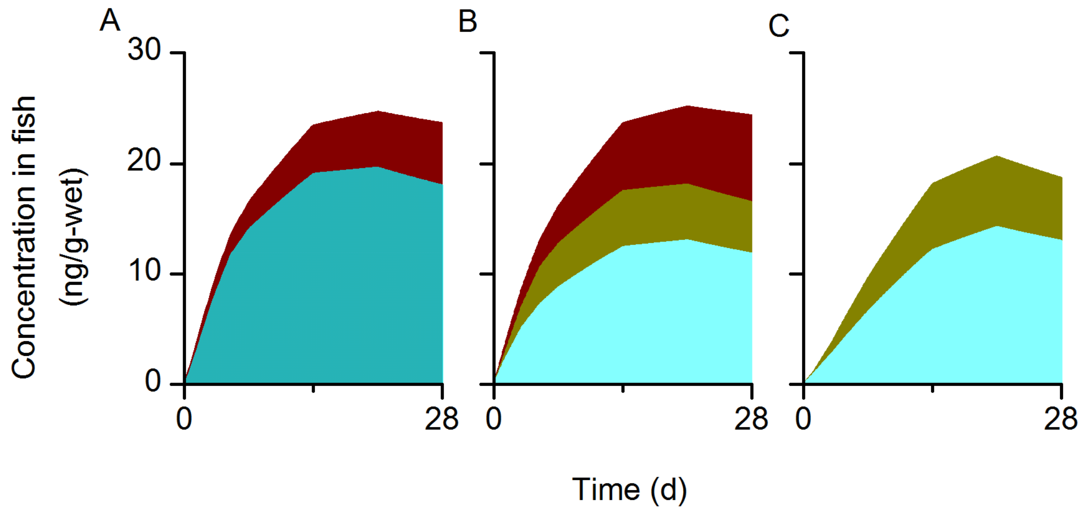

Figure S2. Contributions from multiple exposure media to PFOS concentrations in fish as predicted by kinetic models. Panel A: fish in bottom-sediment-exposure treatment (BST) by Model (I) [Exposure media (from bottom to top): total concentration in water (dissolved + particulate) and bottom sediment]; Panel B: fish in BST by Model (II) [dissolved phase, particulate phase, and bottom sediment]; Panel C: fish in suspended-sediment-exposure treatment (SST) by Model (II) [dissolved phase and particulate phase].

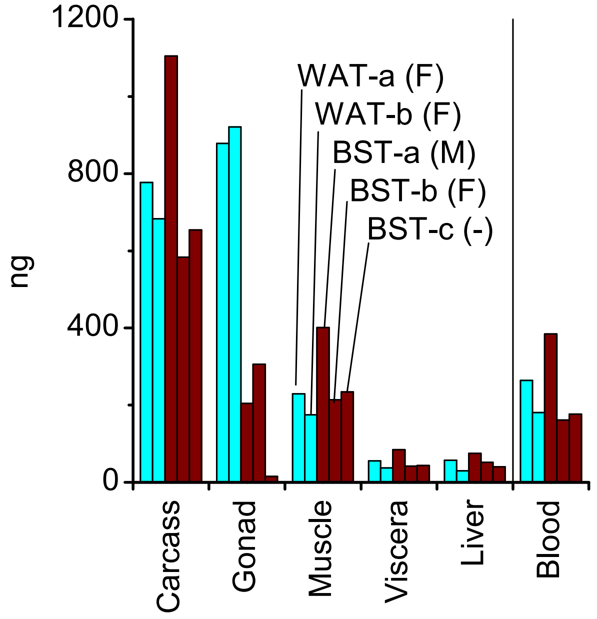

Figure S3. PFOS mass distribution among fish tissues. WAT: water-exposure treatment; BST: bottom-sediment-exposure treatment. Letters a–c indicate individual fish. (F) or (M) indicates female or male judged by gonad observation, and (–) indicates that gender could not be determined because of insufficient gonad development. Viscera do not include the gonad and liver. PFOS mass in each tissue includes that in the blood contained in the tissue.
